# Evaluation of circulating IgG antibodies against *Porphyromonas gingivalis* or its gingipains as serological markers of periodontitis and carriage of the bacterium

**DOI:** 10.1002/JPER.23-0766

**Published:** 2024-06-17

**Authors:** Laura Massarenti, Claus Henrik Nielsen, Anne Katrine Danielsen, Peter Østrup Jensen, Christian Enevold, Christian Damgaard

**Affiliations:** ^1^ Section for Oral Biology and Immunopathology, Department of Odontology, Faculty of Health and Medical Sciences University of Copenhagen Copenhagen Denmark; ^2^ Institute for Inflammation Research, Center for Rheumatology and Spine Diseases, Rigshospitalet Copenhagen University Hospital Copenhagen Denmark; ^3^ Costerton Biofilm Center, Department of Immunology and Microbiology University of Copenhagen Faculty of Health and Medical Sciences Copenhagen Denmark; ^4^ Department of Clinical Microbiology Rigshospitalet Copenhagen Denmark

**Keywords:** biomarker, immunoglobulin, periodontitis, *Porphyromonas gingivalis*

## Abstract

**Background:**

Increasing evidence indicates that periodontitis contributes to systemic low‐grade inflammation. *Porphyromonas gingivalis* is strongly associated with periodontitis, and antibodies against the bacterium may be used as a serological proxy to account for periodontal status, when studying diseases associated with periodontitis. The aim of the present study is to identify an easily accessible and reliable serological biomarker for determination of periodontal status and oral carriage of the bacterium.

**Methods:**

Saliva and serum samples were collected from periodontally healthy controls (*n* = 27), and patients with periodontitis stage II (*n* = 12) or stages III or IV (*n* = 44). Serum levels of immunoglobulin G (IgG) antibodies against intact and fragmented *P. gingivalis*, recombinant gingipains (RgpA and RgpB), and the bacteria *Escherichia coli* and *Capnocytophaga ochracea* as controls were quantified with a multiplex bead‐based assay. *P. gingivalis* was identified in saliva using quantitative polymerase chain reaction (qPCR).

**Results:**

Serum IgG antibodies against *P. gingivalis* whole bacteria were good indicators of periodontitis (area under the curve [AUC]: 0.75, 95% confidence interval [CI]: 0.64–0.85). The same was observed for levels of antibodies against *P. gingivalis* fragments (AUC: 0.78, 95% CI: 0.68–0.88). Likewise, levels of antibodies against *P. gingivalis* whole bacteria or *P. gingivalis* fragments were good indicators of oral carriage of *P. gingivalis* (AUC: 0.92, 95% CI: 0.86–0.98 and AUC: 0.96, 95% CI: 0.92–1, respectively). Conversely, antibodies against recombinant RgpA and RgpB were not good indicators of periodontitis or oral carriage of the bacterium. None of the antibody levels differed significantly between stage II and stage III or IV periodontitis.

**Conclusion:**

Serum IgG antibody levels against heat‐inactivated whole *P. gingivalis* proved to be the preferable biomarker for periodontitis and oral carriage of the bacterium.

## 1 INTRODUCTION

Periodontitis is a multifactorial, chronic inflammatory disease affecting the tooth‐supporting tissues.^1^ The current classification divides the disease into four stages (I–IV) with increasing severity and three grades, A–C, with increasing progression rate.^2^ Periodontitis is very prevalent, and in a large Norwegian cohort of adults, stages I, II, III, and IV affected 13.8%, 41.1%, 15.3%, and 2.3%, respectively, while grades A, B, and C were seen in 5.7%, 60.2%, and 6.2% of individuals, respectively.^3^ However, the prevalence of periodontitis increases with age, and the more severe forms affect primarily persons older than 60 years.^3^ An increasing body of evidence has linked periodontitis to other inflammatory diseases.^4^, ^5^, ^6^, ^7^, ^8^ However, studies focusing on these diseases often lack information on periodontal status and therefore cannot take this parameter into account. Periodontitis case definition relies primarily on measures of clinical attachment loss (CAL) and periodontal pockets detected clinically using a periodontal probe. Probing accuracy and precision rely on multiple factors, such as probe design, probing force, and pocket tissue inflammation, and intraexaminer and interexaminer variation can be considerable even for trained and calibrated clinicians.^9^ Thus, full‐mouth periodontal probing is a time‐consuming process and is therefore not feasible to include in most studies. Hence, a robust biomarker of periodontitis would greatly reduce clinical resource demands and conveniently expand the possibilities of accounting for periodontitis. Reproducible serological assays hold the potential to be an important research tool for determining periodontal status of individuals from whom blood samples are available.

*Porphyromonas gingivalis* is an opportunistic, gram‐negative, anaerobic bacterium, which is strongly associated with periodontitis, but it can also be found, with low prevalence, in periodontally healthy individuals.^10^, ^11^ *P. gingivalis* naturally resides in deep periodontal pockets,^10^ but it can also be found in saliva, supragingival plaque, and other oral mucous membranes including lingual crypts.^11^ Circulating antibodies against the bacterium are common in patients with periodontitis^12^, ^13^, ^14^ and, being stable over time, are regarded as a good marker of previous exposure to the bacterium.^15^ Additionally, *P. gingivalis* has been detected in inflamed joints of patients with rheumatoid arthritis,^16^ in brain tissue of patients with Alzheimer's disease,^17^ and in atherosclerotic plaques.^18^ Moreover, antibodies against *P. gingivalis* and its virulence factors, including arginine gingipain B (RgpB), have been reported to be increased in inflammatory diseases other than periodontitis, including rheumatoid arthritis,^19^, ^20^, ^21^ cardiovascular disease,^22^ Alzheimer's disease,^23^ and kidney disease.^24^ Thus, periodontal status should be accounted for when investigating the etiology of inflammatory diseases.

In the present study, we aimed to identify a robust serological biomarker allowing for the discrimination between periodontal health and periodontitis and between individuals with and without oral carriage of *P. gingivalis*. To this end, we determined the prevalence of *P. gingivalis* in saliva from periodontally healthy individuals and patients with different stages of periodontitis, and measured serum immunoglobulin G (IgG) antibody levels against intact as well as against fragmented bacteria and recombinant RgpA and RgpB.

## 2 MATERIALS AND METHODS

### 2.1 Patients and periodontally healthy controls

This retrospective study was based on samples collected from 89 participants attending the Department of Odontology, University of Copenhagen, from February 2017 to October 2018. It is part of a series of investigations analyzing inflammatory responses and microbial characteristics in patients with periodontitis of different grades and healthy controls, with an original intended sample size of 30 participants in each group.^25^, ^26^, ^27^, ^28^ Medically healthy participants were enrolled prior to periodontal treatment and initially received a diagnosis based on the 1999 criteria for periodontal diseases defined by the World Workshop and American Academy of Periodontology but were later reclassified according to the 2018 criteria for periodontal diseases defined by the American Academy of Periodontology and the European Federation of Periodontology.^2^ Due to lack of saliva samples from four participants (one healthy control and three patients with periodontitis), this study reports data from 27 periodontally healthy controls, 12 participants with periodontitis stage II, 44 participants with periodontitis stage III, and two participants with stage IV. For the analyses, participants in stages III and IV have been grouped. No patients with periodontitis stage I were included in the study. Periodontal examinations and tentative periodontal diagnoses were performed at inclusion by a single examiner (A.K.D.). Diagnosis was subsequently confirmed or changed by an experienced dentist (C.D.) via review of clinical registrations. Periodontitis was defined in accordance with the 2018 classification based on presence of interproximal CAL with bleeding on probing (BOP) on at least two non‐neighboring teeth excluding third molars and were considered inclusion criteria for patients with periodontitis, while grading was based on the percentage of CAL compared with age.

Exclusion criteria were: less than 20 teeth, pregnancy, breastfeeding, antibiotic treatment within the latest 6 months, systemic diseases affecting the immune system such as hematologic anomalies, genetic diseases or syndromes, and any immunosuppressive medication within the latest 2 weeks. All participants gave informed written consent prior to enrollment, and the study was approved by the regional ethical committee (The Capital Region of Denmark, protocol no. H‐1602473) and the Danish Data Authorization (approval no. P‐2019‐18) and registered at ClinicalTrials.gov (no. NCT03225950).

### 2.2 Clinical registrations

Background information of the study population including age, sex, ethnicity, and smoking status was recorded (Table 1). Saliva samples were not available for one healthy control and three patients with periodontitis. Clinical registrations were recorded at six sites per tooth (third molars excluded), including probing pocket depth (PPD), CAL, BOP, plaque index (PI), number of teeth excluding third molars, and number of decayed, missing, or filled teeth.

**TABLE 1:** Study population demographics and clinical data.

Variable	Healthy controls (*n* = 27)	Stage II periodontitis (*n* = 12)	Stages III or IV periodontitis (*n* = 46)	*p* value
Age (mean in years, range)	40 (22–61)	42 (30–55)	42 (20–57)	0.91
Female, *n* (%)	8 (30%)	3 (25%)	13 (28%)	0.96
Current smokers, *n* (%)	3 (11%)	4 (33%)	15 (33%)	0.11
Ethnicity, *n* (%)				
Caucasian	26 (96%)	10 (83%)	41 (89%)	
African	–	1 (8%)	2 (4%)	
Latino	1 (4%)	1 (8%)	3 (7%)	0.67
Grade, *n* (%)				
Grade B	–	8 (67%)	23 (50%)	
Grade C	–	4 (33%)	23 (50%)	<10^−4^
PI in mm (mean, range)	3.6 (0–56.4)	45 (0–80.4)	63 (0–100)	<10^−4^
BOP in % (mean, range)	0.6 (0–15.5)	22.7 (0–76.2)	53.5 (3.6–100)	<10^−4^
PPD in mm (mean, range)	2.3 (1.4–2.7)	2.5 (1.6–3.3)	3.7 (2.5–6.6)	<10^−4^
CAL in mm (mean, range)	1.8 (0.9–2.7)	2.5 (1.8–3.2)	4.1 (2.5–6.6)	<10^−4^
DMFT (mean, range)	5.7 (0–16)	6.8 (0–21)	6.7 (0–20)	<10^−4^
Number of teeth (mean, range)	27.6 (25–28)	27.5 (25–28)	27.1 (21–28)	0.006

*Note*: Mean age between groups was compared with analysis of variance, while the other variables were compared with Kruskal–Wallis test.

Abbreviations: BOP, bleeding on probing; CAL, clinical attachment loss; DMFT, decayed, missing, or filled teeth; PI, plaque index; PPD, probing pocket depth.

### 2.3 Serum and saliva samples

Immediately after the clinical examination, blood and unstimulated saliva samples were collected as previously described.^26^ In brief, serum was obtained from 4 mL of venous blood collected by venipuncture sampling in Vacutainer clot activator tubes* and centrifuged at 350 × *g* for 10 min. Unstimulated saliva samples were collected in Oragene DNA tubes.† Serum and saliva samples were aliquoted and immediately stored at −80°C until further analysis.

### 2.4 qPCR assays for quantitation of *P. gingivalis*

DNA was extracted from 400 μL of saliva with the Maxwell 16 Blood DNA Purification Kit.‡ A previously described in‐house probe‐based quantitative polymerase chain reaction (qPCR) assay for the detection of DNA from *P. gingivalis* was designed using Primer3^29^ to target the highly conserved gene encoding the *P. gingivalis* virulence factor peptidyl arginine deiminase (PPAD) (GenBank: KP862656.1) and purchased from Integrated DNA Technologies.^28^ Sequences of primers and probes for the PPAD qPCR assay are presented in Table S1 in the online *Journal* *of Periodontology*. Testing of the assay performance was carried out with serial dilutions of DNA purified from human saliva or plasma spiked with known amounts of *P. gingivalis* W83 strain as well as with serial dilutions of DNA oligonucleotides corresponding to the exact qPCR product. The latter was run on each plate to generate a standard curve for threshold determination and quantitation of *P. gingivalis* copies. Only samples with cycle threshold (Ct) values corresponding to more than 40 *P. gingivalis* copies (limit of detection of the standard curve) were regarded as positive. Amplification reactions were carried out in a Stratagene Mx3000P thermocycler§ with the following thermal cycling conditions: 3 min at 95°C, 40 cycles at 95°C for 15 s, and 60°C for 1 min. All samples and standard curves, as well as no‐template controls, were run in duplicates. To confirm the results, randomly selected samples (10%) were run twice.

### 2.5 Bacteria and bacterial antigens

*P. gingivalis* strain 2561 (ATCC 33277), a nonpathogenic *Escherichia coli* strain P1 (BAA‐1427), and a *Capnocytophaga ochracea* strain (ATCC 27872) were purchased from American Type Culture Collection (ATCC). Bacteria were resuspended in 500 μL phosphate‐buffered saline (PBS)∥and heat‐inactivated in a 70°C water bath for 1 h. Afterwards bacteria were stained with 5 μM SYTO 60 Red Fluorescent Nucleic Acid Stain¶ and 5 μM SYTO 9 Green Fluorescent Nucleic Acid Stain,# and the bacterial density was determined using an Attune NxT flow cytometer.** Heat‐inactivated bacterial cells were identified with a morphological gating based on forward scatter height and side scatter height, as well as based on the fluorescence emitted at 515–545 nm and at 662–677 nm by the two dyes used for staining. The concentration of bacterial cells was calculated from the number of identified bacteria and counting beads using TrucountTM tubes.†† Recombinant RgpA‡‡ and RgpB§§ were purchased.

### 2.6 Preparation of *P. gingivalis* fragments

*P. gingivalis* fragments were prepared as previously described.^30^ In brief, the three bacterial strains of ATCC 33277, W83, and HG1025, representing serotypes a, b, and c, respectively, were cultured and disintegrated with the use of Lysing Matrix A capsules∥∥ and an intermittent cooling in a FastPrep 24 homogenizer.¶¶ The homogenate was then centrifuged at 20,000 × *g* for 5 min, and the supernatant was stored at −20°C.

### 2.7 Luminex multiplex assay for antibacterial antibodies

About 10^6^ count units of intact bacteria (*P. gingivalis, E. coli*, and *C. ochracea*), 1:500 dilution of the *P. gingivalis* fragments preparation, and 20 μg/mL RgpA or RgpB were coupled to individual xMAP beadsets## using the xMAP Antibody Coupling Kit*** on 2.5 × 10^6^ of each beadset. A blank beadset without antigens was included in the multiplex assay to account for reactivity against the beads per se. In brief, all beadsets were mixed in dilution buffer composed of PBS supplemented with 0.05% Tween 20††† and 1% fetal bovine serum‡‡‡ to reach a final concentration of 25 beads/μL for each beadset. About 50 μL of serum samples diluted 1:80 in dilution buffer were mixed with 50 μL of beadmix and incubated for 1.5 h while shaking. Afterwards, wells were washed three times with 100 μL wash buffer (PBS with 0.05% Tween 20). Biotinylated mouse monoclonal antihuman IgG (Fc‐specific) antibody,§§§ diluted 1:8000 in dilution buffer, was added to the beads and incubated for 1 h, and following three additional washes, the beads were incubated with 100 μL streptavidin phycoerythrin∥∥∥ to a final concentration of 2 μg/mL, for 30 min while shaking. Three additional washes were performed before resuspension of the beads in 100 μL wash buffer and sample acquisition on a Bio‐Plex 200 instrument.¶¶¶ All samples were run on the same plate, and the clinical status was not known to the assay performer.

### 2.8 Statistical analysis

All calculations were made in RStudio Version 2022.12.0+353### using R version 4.2.2.**** Differences in frequencies of oral carriage of *P. gingivalis* across the three groups were evaluated with Fisher's exact test. Linear and logistic regression analyses with correction for age, sex, and smoking status were performed to test associations between log‐transformed antibody levels and periodontal status. In order to determine the ability of anti‐*P. gingivalis* serum antibody levels to differentiate between individuals with and without periodontitis as well as between oral carriers and noncarriers of the bacterium, receiver operating characteristic (ROC) curve analyses were carried out with the pROC package in R. Confidence intervals were calculated with the DeLong method and thresholds were selected based on the maximal obtainable values of the sum of sensitivity and specificity. *p* values <0.05 were considered significant. Graphical representation of the data was performed with GraphPad Prism 9.2 software.††††

## 3 RESULTS

### 3.1 Associations between serum antibody levels and periodontitis

**FIGURE 1:**
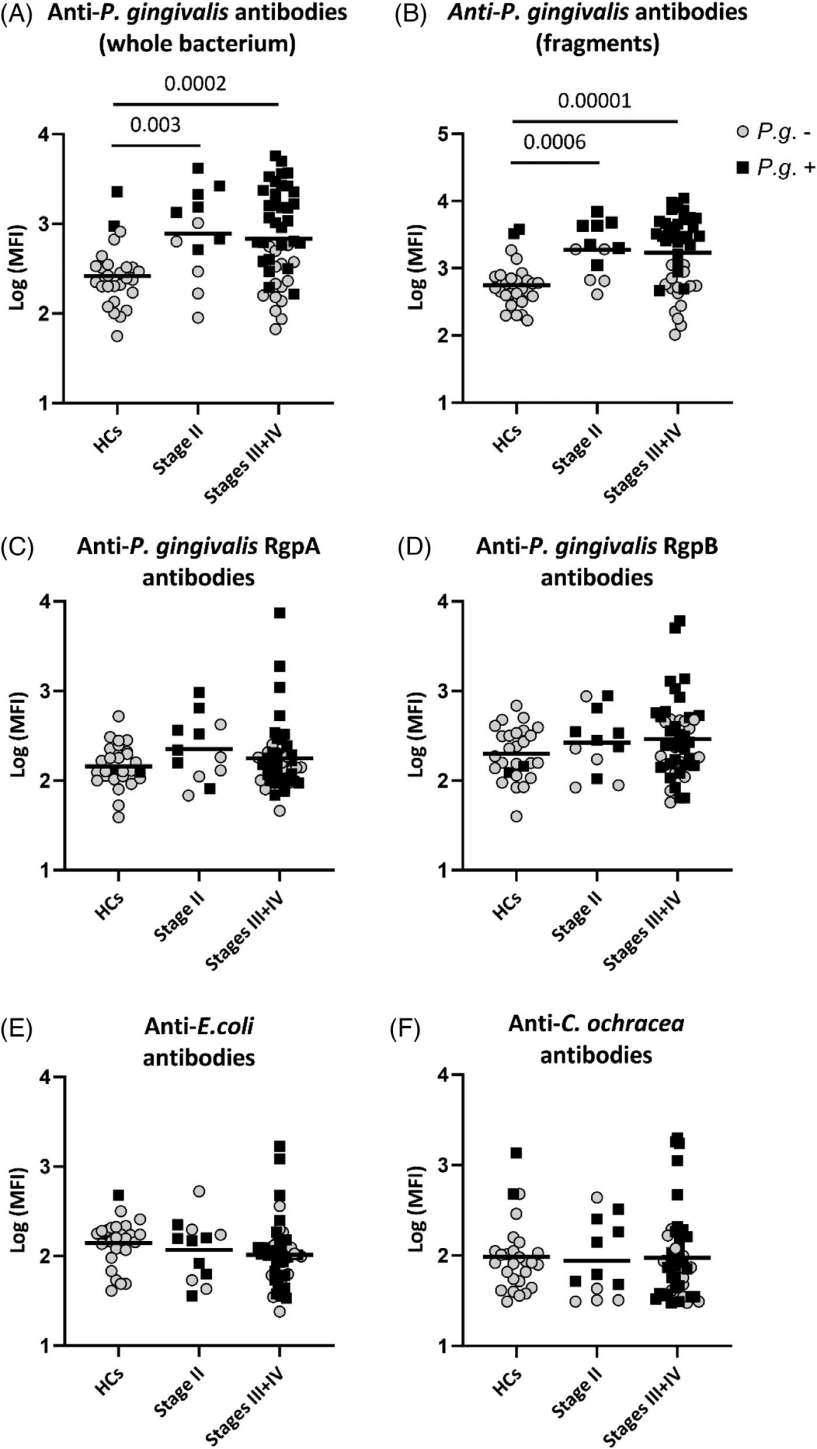
Serum antibody levels against bacteria and recombinant *Porphyromonas gingivalis* gingipains. Antibodies against whole and fragmented bacteria and recombinant gingipains from *P. gingivalis* were measured in sera from 27 periodontally healthy controls (HCs), 12 patients with periodontitis stage II, and 46 patients with periodontitis stages III or IV (stages III + IV) by means of Luminex technology. Log 10 of median fluorescence intensity (MFI) values is shown. The antigens coupled to the beads were as follows: (A) *P. gingivalis* whole bacteria, (B) *P. gingivalis* fragments, (C) recombinant *P. gingivalis* gingipain A (RgpA), (D) recombinant *P. gingivalis* gingipain B (RgpB), (E) *Escherichia coli* whole bacteria, and (F) *Capnocytophaga ochracea* whole bacteria. Black squares and open circles represent individuals positive and negative, respectively, for presence of *P. gingivalis* (*P.g*.) in saliva samples, as determined by quantitative polymerase chain reaction (qPCR). Horizontal bars represent mean values. *p* values were adjusted for age, sex, and current smoking status.

Levels of serum IgG antibodies against *P. gingivalis* whole bacteria were significantly higher in patients with stage II periodontitis and in those with stages III or IV periodontitis than in healthy controls (*p* = 0.003 and *p* = 0.0002 respectively, Figure 1A), while comparable levels were observed between the two periodontitis groups. A similar pattern was observed for antibody levels against fragments derived from *P. gingivalis* (healthy controls vs. stage II, *p* = 0.0006, and healthy controls vs. stages III or IV, *p* = 0.00001, Figure 1B). On the other hand, antibody levels against recombinant RgpA and RgpB from *P. gingivalis* did not differ significantly between healthy controls and the two periodontitis groups (Figure 1C and D).

After logistic regression with adjustment for age, sex, and smoking, levels of antibodies against *P. gingivalis* whole bacteria or fragments were associated with periodontitis independent of stage (*p* < 0.0001 and *p* = 0.0005, respectively), while the same was not observed for antibodies against RgpA or RgpB (*p* = 0.23 and *p* = 0.11, respectively).

Antibody levels against the two control bacteria *E. coli* and *C. ochracea*, which are both gram‐negative rods, were not significantly different between the groups (Figure 1E and F) although patients with stages III or IV periodontitis tended to have lower levels of antibodies against *E. coli* than healthy controls (*p* = 0.07, Figure 1E).

Differences in antibody levels between healthy controls and patients with periodontitis subdivided on the basis of grading are shown in Figure S1 in the online *Journal of Periodontology*.

### 3.2 Serum antibody levels as indicator of periodontitis

ROC curve analysis was performed to determine the ability of antibody levels to discriminate patients with periodontitis at different stages from healthy controls. As presented in Table 2, IgG antibody levels against intact *P. gingivalis* whole bacterium discriminated patients with periodontitis stage II from healthy controls with a sensitivity of 75% and a specificity of 85%, with the determined cutoff at a median fluorescence intensity (MFI) of 476 (area under the curve [AUC]: 0.78, 95% confidence interval [CI]: 0.58–0.97). With the determined cutoff of 355 MFI, we observed a marginally lower sensitivity (70%) and specificity (81%) for the identification of patients with periodontitis stages III or IV (AUC: 0.74, 95% CI: 0.63–0.86). The levels of IgG antibodies against fragmented *P. gingivalis* gave similar results, while the level of IgG antibodies against the two recombinant gingipains (RgpA and RgpB) did not prove able to discriminate between healthy controls and patients with periodontitis of either stage.

**TABLE 2:** Receiver operating characteristic (ROC) curve analyses for antibody levels against selected antigens as markers for periodontitis staging.

	Antigen	AUC	95% CI	Sensitivity	Specificity	Cutoff (MFI)
Stage II	*Porphyromonas gingivalis* whole bacteria	0.78	0.58–0.97	75	85	>476
	*P. gingivalis* fragments	0.85	0.71–0.99	75	85	>979
	*P. gingivalis* RgpA	0.66	0.45–0.87	42	96	>319.5
	*P. gingivalis* RgpB	0.58	0.37–0.80	75	48	>165.5
Stages III + IV	*P. gingivalis* whole bacteria	0.74	0.63–0.86	70	81	>355
	*P. gingivalis* fragments	0.77	0.66–0.87	70	85	>865.5
	*P. gingivalis* RgpA	0.53	0.40–0.67	61	56	>132
	*P. gingivalis* RgpB	0.60	0.47–0.73	35	89	>427.5

Abbreviations: AUC, area under the curve; CI, confidence interval; MFI, median fluorescence intensity.

When combining the two periodontitis groups, levels of antibodies against whole *P. gingivalis* could discriminate between patients and controls with an AUC of 0.75 and a 95% CI of 0.64–0.85. With the selection of the optimal cutoff at 355 MFI, the specificity was 81% and the sensitivity 71%. Similarly, levels of antibodies against *P. gingivalis* fragments could discriminate patients with periodontitis from healthy controls with 85% specificity and 71% sensitivity with a threshold at 865.5 MFI (AUC: 0.78, 95% CI: 0.68–0.88).

### 3.3 *P. gingivalis* oral carriage

Similar to what has previously been reported for participants in this cohort,^28^ *P. gingivalis* DNA was found in saliva from two (7%) healthy controls versus seven (58%) patients with periodontitis stage II (*p* = 0.001) and 31 (67%) patients with periodontitis stages III or IV (*p* < 0.00001).

The presence of the bacteria in saliva was a moderately good discriminator of periodontitis stage II (AUC: 0.75, 95% CI: 0.60–0.91), with 58% sensitivity and 93% specificity. Similarly, patients with periodontitis stages III or IV could be distinguished from healthy controls based on *P. gingivalis* presence in saliva with 67% sensitivity and 93% specificity (AUC: 0.80, 95% CI: 0.71–0.88). Patients with periodontitis, independent of staging, could be distinguished from healthy controls with 66% sensitivity and 93% specificity (AUC: 0.79, 95% CI: 0.71–0.87).

### 3.4 Serum antibody levels as indicator of *P. gingivalis* oral carriage

Additionally, we evaluated the ability of antibody levels against the selected *P. gingivalis* antigens to discriminate between oral carriers and noncarriers of the bacterium independently of periodontal health or disease. Thus, as shown in Table 3, *P. gingivalis* carriers could be distinguished based on antibody levels against intact bacteria, with an AUC of 0.92, a 95% CI of 0.86–0.98, a specificity of 91%, and a sensitivity 83%, with a cutoff of 580 MFI. Moreover, antibodies against fragmented bacteria could provide 100% specificity and 85% sensitivity when using an optimized cutoff level of 1960 MFI (AUC: 0.96, 95% CI: 0.92–1). In contrast, antibody levels against either of the two recombinant gingipains did not discriminate well between carriers and noncarriers of *P. gingivalis*.

**TABLE 3:** Receiver operating characteristic (ROC) curve analyses for antibody levels against selected antigens as markers for salivary carriage of *Porphyromonas gingivalis*.

Antigen	AUC	95% CI	Sensitivity	Specificity	Cutoff (MFI)
*P. gingivalis* whole bacteria	0.92	0.86–0.98	83	91	>580
*P. gingivalis* fragments	0.96	0.92–1	85	100	>1960
*P. gingivalis* RgpA	0.60	0.47–0.72	33	93	>285
*P. gingivalis* RgpB	0.61	0.49–0.73	33	96	>504

Abbreviations: AUC, area under the curve; CI, confidence interval; MFI, median fluorescence intensity.

After logistic regression with adjustment for age, sex, and smoking, levels of antibodies against *P. gingivalis* whole bacteria or fragments were associated with oral carriage of the bacterium (*p* < 0.00001 and *p* < 0.00001, respectively). The same was also observed for antibodies against RgpA or RgpB, although to a minor extent (*p* = 0.03 and *p* = 0.03, respectively).

## 4 DISCUSSION

In this study, we determined the validity of circulating antibodies against whole and fragmented *P. gingivalis* and antibodies against recombinant gingipains as diagnostic biomarkers for periodontitis in an otherwise medically healthy cohort. Likewise, we evaluated the ability of the selected biomarker to indicate oral carriage of the bacterium.

The levels of circulating anti‐*P. gingivalis* IgG antibodies against whole or fragmented bacteria were increased in patients with periodontitis compared to healthy controls. However, individual levels of antibodies did not have prognostic value since no significant statistical difference was found between antibody levels in different disease stages or grades in the tested cohort. While we observed no significant differences in antibody levels to one of the negative control bacteria, *C. ochracea*, between the three groups of participants, antibody levels against the other negative control bacterium, *E. coli*, tended to be lower in patients with stage III or IV periodontitis than in healthy controls. However, the relevance of *E. coli* in the pathogenesis of periodontitis is controversial.

From a practical standpoint and in terms of reproducibility, usage of recombinant gingipains as antigens in the immunoassay would be preferable compared to the usage of whole or fragmented bacteria. However, while the levels of antibodies against recombinant RgpA and RgpB tended to be increased in the two patient groups, we did not find a statistically significant difference between those levels and the corresponding levels in healthy controls. This is in contrast to previous reports showing increased levels of antibodies against RgpB in individuals with periodontitis.^4^, ^5^, ^21^ These discrepancies may be due to differences in the purification processes or in the expression host for recombinant RgpB^31^, ^32^ and/or due to our study being underpowered compared to other studies. On the other hand, in a study by de Vries et al., antibodies against RgpB were particularly elevated in patients with severe periodontitis, also in comparison to patients with mild periodontitis. Furthermore, in the same study, the antibody levels were good discriminators of periodontitis in one of the cohorts studied, while the same was not true for the other cohort.^4^ Contrasting results on the validity of antibodies to gingipains as indicators of periodontitis were found in another study showing that antibodies to hemagglutinin domains of RgpA, but not those to the catalytic domains of the same gingipain, were increased in patient with periodontitis.^33^

ROC curve analyses revealed that the levels of antibodies against intact *P. gingivalis* (representing serotype a) were useful for discrimination of individuals with and without periodontitis, with a sensitivity similar to that of the level of antibodies against fragmented *P. gingivalis* (representing serotypes a, b, and c). Our assay for antibodies against intact *P. gingivalis* shows similar diagnostic performance to previously reported assays,^13^, ^14^ but the results obtained in this study suggest that it is not worthwhile to adapt the more cumbersome preparation of the fragmented bacterial antigens from several serotypes. The use of antibodies against *P. gingivalis* as indicator of periodontitis has been proposed previously, but the results have been contradictive, presumably due to differences in choice of antigen and the severity of disease of patients included in the studies. With few exceptions, RgpB and bacterial fragments have been shown to be good indicators of periodontitis,^4^, ^5^, ^13^, ^21^ while antibodies to the other gingipains or other virulence factors like PPAD were not consistently found to be elevated in patients with periodontitis.^21^, ^33^

Comparably to what has been reported in other studies,^12^, ^14^, ^25^ *P. gingivalis* was found in more than 60% of patients with periodontitis versus only 7% of healthy controls, as determined by the presence of DNA encoding PPAD from the bacterium in saliva. The presence of *P. gingivalis* in saliva thus proved to be a putative tool for identification of patients with periodontitis in studies when saliva samples collected for other purposes are available. Furthermore, the serum level of both *P. gingivalis* antibodies and antibodies against *P. gingivalis* fragments appeared to be reliable markers of current infection with *P. gingivalis*, which may be a useful tool in situations where serum or plasma but not saliva samples are available.

The results presented here are obtained from a limited number of samples (*n* = 85) and should therefore be validated in larger cohorts. Nevertheless, serum levels of antibodies against *P. gingivalis* (intact as well as fragmented) proved able to discriminate between individuals with and without periodontitis. Such antibody measurements may be useful in studies on the etiology of, for example, rheumatoid arthritis, systemic lupus erythematosus, or cardiovascular disease, where periodontal examinations are not routinely performed. Of note, elevated levels of antibodies against *P. gingivalis* have been reported in patients with rheumatoid arthritis^19^, ^20^, ^21^ or systemic lupus erythematosus,^34^ as well as in individuals with cardiovascular disease.^22^, ^35^

Notably, usage of antibodies against intact *P. gingivalis* as biomarker for determination of periodontal status is likely more reliable than questionnaire‐based data, which might be subject to recall bias. Indeed, levels of antibodies against intact *P. gingivalis* used in this study seemed to have better diagnostic power than a questionnaire‐based determination of periodontal status.^36^ On the other hand, it may be worthwhile to combine the serological data with information obtained from questionnaires in order to increase sensitivity further. However, these are rarely available, and therefore additional serological biomarkers could be chosen instead. Nevertheless, the methods described in this paper are intended for use in situations where information on periodontal status is lacking, and dental examinations or microbiological analyses are not possible, in order to evaluate the potential confounding effect of periodontitis. In such a scenario, a high specificity might be preferable to a high sensitivity with the tradeoff of observing a smaller confounding effect of periodontitis, while lowering the number of false positives to the minimum.

Taken together, the results presented in this study suggest that IgG antibodies against heat‐inactivated *P. gingivalis* whole bacteria or fragmented *P. gingivalis* may serve as serological biomarkers for periodontitis and oral carriage of the bacterium. When considering assay reproducibility, preparation procedure, and costs, the preferable serological biomarker for periodontitis and for oral carriage of the bacterium is heat‐inactivated *P. gingivalis* whole bacteria.

## AUTHOR CONTRIBUTIONS

Laura Massarenti, Christian Enevold, and Claus H. Nielsen conceived and designed the experiments. Anne Katrine Danielsen and Christian Damgaard recruited the participants and collected the data. Christian Damgaard diagnosed the participants according to the 2017 classification. Laura Massarenti performed the experiments and analyzed the data. Peter Østrup Jensen aided with bacteria heat inactivation and counting, and Christian Enevold aided with statistical analysis. Laura Massarenti drafted the manuscript and designed the figures. Claus H. Nielsen, Christian Enevold, Christian Damgaard, Anne Katrine Danielsen, and Peter Østrup Jensen assisted in writing the manuscript. All authors gave their final approval and agreed to be accountable for all aspects of the work.

## CONFLICT OF INTEREST STATEMENT

All authors declare no financial conflicts of interest.

## ETHICS STATEMENT

This study was approved by the ethics board of the regional ethical committee (The Capital Region of Denmark, protocol no. H‐1602473) and the Danish Data Authorization (approval no. P‐2019‐18) and was conducted in accordance with the Helsinki Declaration of 1975, as revised in 2013.

## Supporting information

Supporting Information

Supporting Information

## Data Availability

The datasets are archived at Rigshospitalet, Copenhagen University Hospital, in line with the Danish Data Authorization (approval no. P‐2019‐18). Access to all data will be shared upon reasonable request (chrd@sund.ku.dk).

## Footnotes

* cat: 369032, Becton Dickinson (BD) Biosciences.

† cat: OG‐500, DNA Genotek Inc.

‡ cat: AS1020, Promega.

§ Agilent Technologies.

∥ cat: 14190144, Gibco.

¶ cat: S11342, Thermo Fisher Scientific.

# cat: S34854, Thermo Fisher Scientific.

** Thermo Fisher Scientific.

†† cat: 340334, BD Biosciences.

‡‡ cat: CSB‐EP338957PQP, Cusabio.

§§ cat: CSB‐EP310587EYA(A4), Cusabio.

∥∥ cat: 116910050, MP Biomedicals.

¶¶ MP Biomedicals.

## Luminex Corporation.

*** Luminex Corporation.

††† cat: P1379, Dako.

‡‡‡ cat: F9665, Sigma‐Aldrich.

§§§ cat: AB3773, Sigma‐Aldrich.

∥∥∥ cat: PJ31S, ProZyme.

¶¶¶ Bio‐Rad.

### RStudio Inc.

**** R Foundation for Statistical Computing.

†††† GraphPad software Inc.
