# Supplementary material for: Evaluation of circulating IgG antibodies against Porphyromonas gingivalis or its gingipains as serological markers of periodontitis and carriage of the bacterium
Source: J Periodontol. 2024 Jun 17;96(2):119–28. doi: 10.1002/JPER.23-0766 (PMC11866731; doi:10.1002/JPER.23-0766)
Supplement: Supplementary file 1 — Supporting Information [file JPER-96-119-s002.pdf]

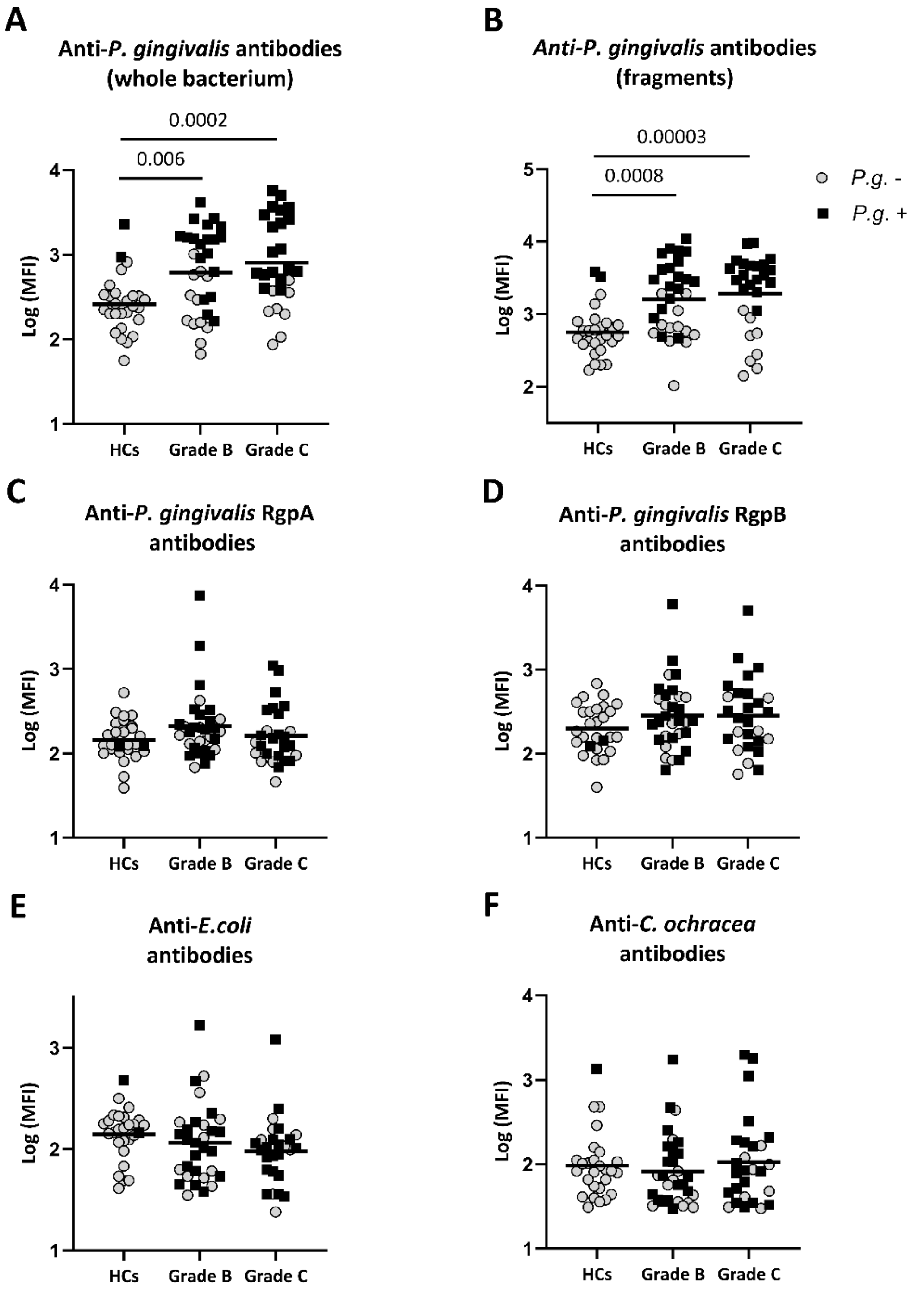

Supplementary Figure 1: Serum antibody levels against bacteria and recombinant *P. gingivalis* gingipains. Antibodies against whole and fragmented bacteria and recombinant gingipains from *P. gingivalis* were measured in sera from 27 periodontally healthy controls (HCs), 31 patients with periodontitis grade B and 27 patients with periodontitis grade C by means of Luminex technology. Log 10 of median fluorescence intensity (MFI) values is shown. The antigens coupled to the beads were as follows: **A)** *P. gingivalis* whole bacteria, **B)** *P. gingivalis* fragments, **C)** recombinant *P. gingivalis* gingipain A (RgpA), **D)** recombinant *P. gingivalis* gingipain B (RgpB), **E)** *E. coli* whole bacteria and **F)** *C. ochracea* whole bacteria. Black squares and open circles represent individuals positive and negative, respectively, for the presence of *P. gingivalis* (*P.g.*) in saliva samples, as determined by qPCR. Horizontal bars represent mean values. *P* values have been adjusted for age, sex, and current smoking status.
