# Supplementary material for: Evaluation of circulating IgG antibodies against Porphyromonas gingivalis or its gingipains as serological markers of periodontitis and carriage of the bacterium
Source: J Periodontol. 2024 Jun 17;96(2):119–28. doi: 10.1002/JPER.23-0766 (PMC11866731; doi:10.1002/JPER.23-0766)
Supplement: Supplementary file 2 — Supporting Information [file JPER-96-119-s001.docx]

| **Supplementary table 1:** Sequences of primers and probes for the *P. gingivalis* PPAD qPCR assay. | | |
| --- | --- | --- |
|  |  | **Sequence** |
| ***P. gingivalis* PPAD** | **Forward primer** | 5'- CAA ACG CAA ATG CAA GCA GAC C -3' |
|  | **Reverse primer** | 5'- GAA CGG GTA GCG TAC CAA AAC G -3' |
|  | **Internal oligo** | 5'- AAT CCC CCT GCA GGT CCT GTG CGT GCT -3' |
